# Synthesis of Novel 1-[(2,6-Dichloro-4-trifluoromethyl)phenyl]-3-aryl-1*H*-pyrazole-4-carbaldehydes

**DOI:** 10.3390/molecules15107472

**Published:** 2010-10-25

**Authors:** Huanan Hu, Changhua Ge, Lisheng Ding, Anjiang Zhang

**Affiliations:** 1 College of Pharmaceutical and Chemical Engineering, Taizhou University, Linhai 317000, China; E-Mail: gechhua@163.com; 2 Chengdu Institute of Biology, Chinese Academy of Sciences, Chengdu 610041, China; E-Mail: lsding@cib.ac.cn; 3 Ningbo Institute of Material Technology and Engineering, Chinese Academy of Sciences, Ningbo 315211, China

**Keywords:** Vilsmeier-Haack reagent, phenylhydrazine, phenylhydrazone, 1*H*-pyrazole-4-carbaldehyde

## Abstract

A series of novel 1-[(2,6-dichloro-4-trifluoromethyl)phenyl]-3-aryl-1*H*-pyrazole-4-carbaldehydes **6a-i** were synthesized using the Vilsmeier-Haack reagent. The structures of all the title compounds have been confirmed by elemental analysis, ^1^H-NMR and ^13^C-NMR and in addition, the structure of intermediate **5b** was investigated by X-ray crystallography.

## 1. Introduction

The application of the Vilsmeier-Haack (VH) reagent (POCl_3_/DMF) for formylation of a variety of both aromatic and heteroaromatic substrates is well documented [1]. Besides this, the reagent has also been extensively used for effecting various chemical transformations with other classes of compounds. Many of these reactions have led to novel and convenient routes for the synthesis of various heterocyclic compounds [2]. A notable example that finds significant application in heterocyclic chemistry is the synthesis of 4-formylpyrazoles from the double formylation of hydrazones with the VH reagent [3,4]. These observations, coupled with the recent developments in the simple synthesis of pyrazole derivatives [5], especially 4-functionalized 1,3-diphenylpyrazoles as antibacterial [6], anti-inflammatory [7], antiparasitic [8], and antidiabetic drugs [9], prompted us to undertake the synthesis of pyrazole-4-carbaldehyde derivatives using the VH reagent. 

It has been known for some time that fluorine atoms can lead to unexpected biological activity results due to the special properties of the fluorine atom, such as the high electronegativity of fluorine and the high carbon-fluorine bond energy [10]. As a consequence, trifluoromethyl-containing molecules have been found considerable utilization in the agrochemical and pharmaceutical industries [10,11,12], for example, pyrazole derivatives bearing trifluoromethyl groups, such as fipronil and analogs [13,14] are widely used phenylpyrazole insecticides applied in granular or bait form for residential and commercial control of turf grass pests, and celecoxib [15], widely prescribed to treat acute or chronic inflammation by providing symptomatic pain relief, are examples of such compounds. In this paper, we show a simple and efficient synthetic method using the VH reagent [4,16,17] that affords a series of novel 1-[(2,6-dichloro-4-trifluoromethyl)phenyl]-3-aryl-1*H*-pyrazole-4-carbaldehydes.

## 2. Results and Discussion

**Scheme 1:**
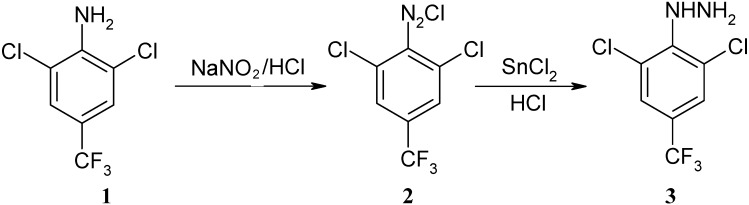
Synthesis of phenylhydrazine **3**.

Phenylhydrazine **3** was synthesized from 2,6-dichloro-4-trifluoromethylphenylamine (**1**) through diazotization, reduction and hydration (Scheme 1). The reducing reagent plays an important role in the reaction. When the intermediate **2** was reduced by SnCl_2_, an almost quantitative yield of product was obtained. Use of Na_2_S_2_O_3_ or Zn as reducing reagents led to lower yields.

**Scheme 2:**
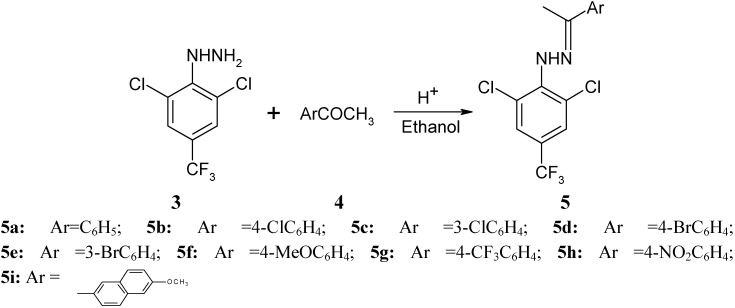
Synthesis of phenylhydrazones **5**.

Phenylhydrazones **5** were next synthesized in almost quantitative yields by the reaction between the phenylhydrazine **3** and 3 or 4-substituted aryl methyl ketones, regardless of whether the ketones contained electron-withdrawing or electron-donating groups (Scheme 2).

### X-ray diffraction

**Figure 1:**
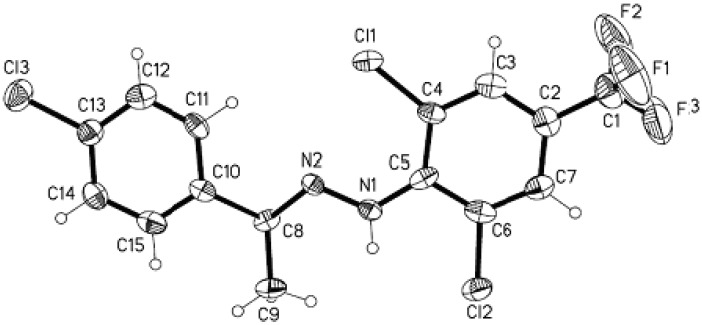
ORTEP drawing of the compound **5b** showing the atom numbering scheme.

To verify the structural assignment compound **5b** was selected as an example for an X-ray diffraction study (Figure 1 and Table 1). The purified product **5b** was dissolved in 50 % ethanol/acetone (1:1 v/v) and kept at room temperature for 5 days and single crystals of **5b** were thus formed. CCDC 685556 contains the supplementary crystallographic data for this comound [18].

**Table 1:** Crystal data and summary of data collection and structure refinement.

Compound	C_15_ H_10_ Cl_3_ F_3_ N_2_
Color	Colorless
Formula weight	381.60
Crystal system	Monoclinic
Temperature,°	25(298K)
Cell constants	
a (Å)	16.8770(7)
b (Å)	8.0054(8)
c (Å)	24.119(2)
α (˚)	90
β (˚)	99.654(2)
γ (˚)	90
Volume (Å^3^)	3212.5(4)
Formula units	8
Calculated density (g/cm^-3^)	1.578
F(000)	1536
Absorption coefficient, mμ^-3^	0.599
Limiting indices	-20<=h<=16, -7<=k<=9, -27<=l<=28
Reflections collected / unique	8110 / 2841 [R(int) = 0.0402]
Absorption correction	Semi-empirical from equivalents
Max. and min. transmission	0.9370 and 0.8314
Refinement method	Full-matrix least-squares on F^2^
Data / restraints / parameters	2841 / 0 / 208
Goodness-of-fit on F^2^	1.015
Final R indices	R_1_ = 0.1641, wR_2_ = 0.5152
Largest diff. peak and hole (e Å^-3^)	0.869 and -0.919

**Scheme 3:**
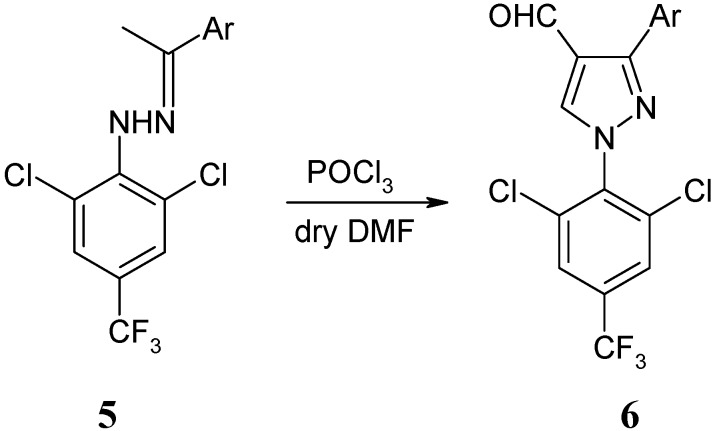
Synthesis of 1*H*-pyrazole-4-carbaldehydes **6**.

The Vilsmeier cyclization of hydrazones could provide an efficient route for the preparation of 1*H* -pyrazole-4-carbaldehydes. The reaction was carried out at 80–90 °C for 4 h using 3 equiv. of the VH reagent, affording a series of novel 1-[(2,6-dichloro-4-trifluoromethyl)phenyl]-3-aryl-1*H*-pyrazole-4-carbaldehydes in good yields (Scheme 3). The results are shown in Table 2. 1*H*-Pyrazole-4-carbaldehydes **6** cannot be obtained if the DMF used is not anhydrous.

**Table 2:** Synthesis of 1*H*–pyrazole-4-carbaldehydes.

Entry	Ar	Products(**6**)	Yield(%) *^a^*
1	C_6_H_5_	**6a**	89
2	4-ClC_6_H_4_	**6b**	88
3	3-ClC_6_H_4_	**6c**	83
4	4-BrC_6_H_4_	**6d**	86
5	3-BrC_6_H_4_	**6e**	82
6	4-MeOC_6_H_4_	**6f**	83
7	4-CF_3_C_6_H_4_	**6g**	86
8	4-NO_2_C_6_H_4_	**6h**	85
9	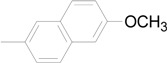	**6i**	81

*^a^* Isolated yields with regard to the quantity of hydrazones **5**

## 3. Experimental

### 3.1. General

All melting points were determined on an XT-4A apparatus and are uncorrected. TLC was performed using precoated silica gel GF_254_ (0.25mm), column chromatography was performed using silica gel (200–300 mesh). The ^1^H- and ^13^C-NMR spectra were measured at 25 °C at 300 and 75 MHz, respectively, on a Bruker Advance 300 spectrometer, using TMS as internal standard. *J*-values are given in Hz. The IR spectra were taken on a Bruker Vector 55 spectrometer. Elemental analyses were carried out with an EA 1112 elemental analyzer. All the reagents used were AR grade.

### 3.2. Synthesis of (2,6-Dichloro-4-trifluoromethyl)phenylhydrazine *(**3**)*

2,6-Dichloro-4-trifluoromethylphenylamine (**1**, 1.73 g, 7.5 mmol) was dissolved in concentrated HCl (10 mL) and water (10 mL) and cooled to 0 °C, sodium nitrite (0.62 g, 9.0 mmol) was added and the yellow solution was stirred at 0 °C for 2 h to get a solution of diazotizated compound **2**. SnCl_2_ (2.85 g, 15 mmol) was dissolved in concentrated HCl (10 mL) and cooled to 7 °C, then the diazotizated solution of **2** was added slowly dropwise. After 1 h of reaction, the precipitate was filtered, washed with water and air-dried. The precipitate was neutralized by 25% NaOH in water to pH 8, the light yellow sediment was collected and air-dried to afford a brown solid **3** (1.53 g, 83%), m.p. 65–67 °C. ^1^H-NMR (CDCl_3_) δ: 7.52 (s, 2H, Ar-H), 5.85 (s, 1H, NH), 3.86 (br, 2H, NH_2_); ^13^C- NMR (CDCl_3_) δ: 137.1, 132.5 (q, *J* = 33.8 Hz), 130.3, 123.8, 122.3 (q, *J* = 271.8 Hz); IR (KBr) ν: 3517, 3418, 1622, 1583, 1492, 1329, 1281, 1144, 883 cm^-1^.

### 3.3. General procedure for synthesis of N-(2,6-Dichloro-4-trifluoromethyl)phenyl-N’-(1-phenyl-ethylidene) hydrazines ***5a-i***

To a solution of compound **3** (12 mmol) in absolute C_2_H_5_OH (30 mL) was added substituted acetophenone **4** (10 mmol). Two drops of concentrated HCl were added to a stirred solution, and the mixture was refluxed for 1 h. The solid obtained on cooling was filtered, dried and recrystallized from C_2_H_5_OH to give compounds **5a-i**.

*N-(2,6-Dichloro-4-trifluoromethyl)phenyl-N’-(1-phenylethylidene)hydrazine* (**5a**): White solid, m.p. 90–91 °C; ^1^H-NMR (CDCl_3_) δ: 7.82 (d, *J* = 3.6 Hz, 2H, N’-Ar-H), 7.67 (s, 1H, NH), 7.58 (s, 2H, N-Ar-H), 7.34–7.42 (m, 3H, N’-Ar-H), 2.35 (s, 3H, CH_3_); ^13^C-NMR (CDCl_3_) δ: 147.2, 140.8, 136.1, 132.5 (q, *J* = 33.8 Hz), 127.3, 126.8, 126.0, 125.8, 123.8, 122.3 (q, *J* = 271.8 Hz), 11.7; IR (KBr) ν: 3350, 3072, 2927, 1603, 1538, 1492, 1390, 1351, 1311, 1114, 1009, 887 cm^-1^.

*N-(2,6-Dichloro-4-trifluoromethyl)phenyl-N’-[1-(4-chlorophenyl)ethylidene]hydrazine* (**5b**): White solid, m.p. 118–120 °C; ^1^H-NMR (CDCl_3_) δ: 7.68–7.76 (m, 3H, N’-Ar-H and NH), 7.58 (s, 2H, N-Ar-H), 7.34–7.37 (m, 2H, N’-Ar-H), 2.33 (s, 3H, CH_3_); ^13^C NMR (CDCl_3_) δ: 146.1, 140.7, 136.3, 134.5 (q, *J* = 33.5 Hz), 128.3, 126.7, 126.2, 126.0, 123.9, 121.8 (q, *J* = 271.8 Hz), 11.8; IR (KBr) ν: 3360, 3070, 2924, 1605, 1532, 1490, 1392, 1352, 1303, 1114, 1004, 884 cm^-1^.

*N-(2,6-Dichloro-4-trifluoromethyl)phenyl-N’-[1-(3-chlorophenyl)ethylidene]hydrazine* (**5c**): White solid, m.p. 118–120 °C; ^1^H-NMR (CDCl_3_) δ: 7.83 (s, 1H, N’-Ar-H), 7.71–7.76 (m, 2H, N’-Ar-H and NH), 7.58 (s, 2H, N-Ar-H), 7.36–7.40 (m, 2H, N’-Ar-H), 2.32 (s, 3H, CH3); ^13^C-NMR (CDCl_3_) δ: 146.1, 140.7, 136.3, 133.5 (q, *J* = 33.3 Hz), 129.2, 128.3, 127.5, 126.7, 126.1, 125.6, 123.9, 122.1 (q, *J* = 271.8 Hz), 12.1; IR (KBr) ν: 3358, 3073, 2926, 1603, 1536, 1489, 1390, 1352, 1313, 1108, 1011, 886 cm^-1^.

*N-(2,6-Dichloro-4-trifluoromethyl)phenyl-N’-[1-(4-bromophenyl)-ethylidene]hydrazine* (**5d**): White solid, m.p. 114–115 °C; ^1^H-NMR (CDCl_3_) δ: 7.65–7.69 (m, 3H, N’-Ar-H and NH), 7.58 (s, 2H, N-Ar-H), 7.48–7.52 (m, 2H, N’-Ar-H), 2.33 (s, 3H, CH_3_); ^13^C-NMR (CDCl_3_) δ: 146.5, 141.7, 134.3, 132.5 (q, *J* = 33.5 Hz), 127.6, 127.1, 126.7, 125.6, 123.9, 121.9 (q, *J* = 271.8 Hz), 11.7; IR (KBr) ν: 3362, 3074, 2928, 1606, 1540, 1495, 1393, 1354, 1308, 1106, 1012, 887 cm^-1^.

*N-(2,6-Dichloro-4-trifluoromethyl)phenyl-N’-[1-(3-bromophenyl)ethylidene]hydrazine* (**5e**): White solid, m.p. 114–115 °C; ^1^H-NMR (CDCl_3_) δ: 7.94 (s, 1H, N’-Ar-H), 7.70–7.75 (m, 2H, N’-Ar-H and NH), 7.58 (s, 2H, N-Ar-H), 7.46–7.49 (m, 2H, N’-Ar-H), 2.32 (s, 3H, CH_3_); ^13^C-NMR (CDCl_3_) δ: 147.1, 141.9, 139.3, 132.3 (q, *J* = 33.5 Hz), 128.3, 127.6, 127.1, 126.7, 126.0, 124.6, 123.9, 121.9 (q, *J* = 271.8 Hz), 11.9; IR (KBr) ν: 3360, 3072, 2924, 1600, 1538, 1492, 1390, 1356, 1307, 1115, 1019, 889 cm^-1^.

*N-(2,6-Dichloro-4-trifluoromethyl)phenyl-N’-[1-(4-methoxyphenyl)ethylidene]-hydrazine* (**5f**): White solid, m.p. 89–90 °C; ^1^H-NMR (CDCl_3_) δ: 7.81 (s, 1H, N’-Ar-H), 7.79 (s, 1H, N’-Ar-H), 7.67 (s, 1H, NH), 7.58 (m, 2H, N-Ar-H), 6.73–6.89 (m, 2H, N’-Ar-H), 3.83 (s, 3H, OCH_3_), 2.32 (s, 3H, CH_3_). ^13^C-NMR (CDCl_3_) δ: 158.1, 147.5, 136.2, 133.6 (q, *J* = 33.6 Hz), 127.8, 126.2, 125.6, 124.7, 121.9 (q, *J* = 270.2 Hz), 114.2, 55.1, 11.7; IR (KBr) ν: 3355, 3072, 2925, 1603, 1530, 1489, 1391, 1351, 1307, 1112, 1016, 887 cm^-1^.

*N-(2,6-Dichloro-4-trifluoromethyl)phenyl-N’-[1-(4-trifluoromethylphenyl)-ethylidene]hydrazine* (**5g**): White solid, m.p. 165–166 °C; ^1^H-NMR (CDCl_3_) δ: 7.91 (d, *J* = 8.4 Hz, 2H, N’-Ar-H), 7.78 (s, 1H, NH), 7.59–7.65 (s, 4H, N’ and N-Ar-H), 2.36 (s, 3H, CH_3_); ^13^C-NMR (CDCl_3_) δ: 145.5, 144.7, 133.5 (q, *J* = 32.6 Hz), 130.4 (q, *J* = 32.1 Hz), 126.3, 125.8, 125.2, 124.6, 124.4, 123.2 (q, *J* = 270.2 Hz), 121.8 (q, *J* = 272.6 Hz), 11.9; IR (KBr) ν: 3351, 3070, 2926, 1601, 1535, 1490, 1389, 1350, 1313, 1116, 1021, 889 cm^-1^.

*N-(2,6-Dichloro-4-trifluoromethyl)phenyl-N’-[1-(4-nitrophenyl)ethylidene]hydrazine* (**5h**): Yellowish solid, m.p. 146–147 °C; ^1^H-NMR (CDCl_3_) δ: 8.24 (d, *J* = 6.0 Hz, 2H, N’-Ar-H), 7.88–7.97 (m, 3H, N’-Ar-H and NH), 7.60 (s, 2H, N-Ar-H), 2.38 (s, 3H, CH_3_); ^13^C-NMR (CDCl_3_) δ: 147.5, 144.3, 143.9, 140.4, 132.8 (q, *J* = 33.6 Hz), 126.5, 125.1, 124.7, 123.6, 120.9 (q, *J* = 272.6 Hz), 11.8; IR (KBr) ν: 3320, 3073, 2926, 1583, 1513, 1393, 1339, 1307, 1117, 890 cm^-1^.

*N-(2,6-Dichloro-4-trifluoromethyl)phenyl-N’-[1-(6-methoxy-naphthalen-2-yl)-ethylidene]hydrazine* (**5i**): White solid, m.p. 191–193 °C; ^1^H-NMR (CDCl_3_) δ: 8.11 (d, *J* = 6.2 Hz, 1H, N’-Ar-H), 8.00 (s, 1H, N’-Ar-H), 7.71–7.78 (m, 3H, N’-Ar-H and NH), 7.58 (s, 2H, N-Ar-H), 7.14-7.17 (m, 2H, N’-Ar-H), 3.84 (s, 3H, OCH_3_), 2.43 (s, 3H, CH_3_); ^13^C-NMR (CDCl_3_) δ: 157.9, 147.5, 134.6, 133.2 (q, *J* = 32.5 Hz), 129.6, 128.2, 126.7, 126.2, 126.1, 125.3, 124.8, 123.8, 123.7, 121.7 (q, *J* = 271.6 Hz), 118.7, 105.5, 55.0, 11.7; IR (KBr) ν: 3353, 3069, 2938, 1602, 1528, 1487, 1390, 1346, 1305, 1127, 1026, 889 cm^-1^.

### 3.4. General procedure for synthesis of 1-[(2,6-dichloro-4-trifluoromethyl)phenyl]-3-aryl-1H-pyrazole-4- carbaldehydes ***6a-i***

POCl_3_ (0.5 g, 3.0 mmol) was added dropwise to an ice-cold stirred solution of hydrazone **5** (1.0 mmol) in dry DMF (4 mL), the reaction mixture was allowed to attain room temperature and then heated at 80 °C for 4 h. The resulting mixture was poured onto crushed ice, neutralized with dilute sodium hydroxide and left standing overnight. The pale yellow precipitate obtained was purified by flash column chromatography with ethyl acetate–petroleum ether mixture to yield the products **6a-i.**

*1-[(2,6-Dichloro-4-trifluoromethyl)phenyl]-3-phenyl-1H-pyrazole-4-carbaldehyde* (**6a**): White solid, m.p. 147–149 °C; ^1^H-NMR (CDCl_3_) δ: 10.11 (s, 1H, CHO), 8.22 (s, 1H, pyrazole H), 7.79–7.83 (m, 4H, 1 and 3-Ar-H), 7.27–7.54 (m, 3H, 3-Ar-H); ^13^C-NMR (CDCl_3_) δ: 182.4, 153.8, 137.8, 135.0, 133.9 (q, *J* = 33.8 Hz), 129.0, 126.1, 125.8, 125.3, 124.6, 123.7, 122.6, 121.9 (q, *J* = 271.8 Hz); IR (KBr) ν: 3107, 3062, 2843, 1695, 1602, 1531, 1403, 1310, 1131, 850, 719 cm^-1^; Anal. Calcd. for C_17_H_9_Cl_2_F_3_N_2_O: C, 53.01; H, 2.36; N, 7.27. Found: C, 53.30; H, 2.45; N, 7.33%.

*1-[(2,6-Dichloro-4-trifluoromethyl)phenyl]-3-(4-chlorophenyl)-1H-pyrazole-4-carbaldehyde* (**6b**): White solid, m.p. 108–110 °C; ^1^H-NMR (CDCl_3_) δ: 10.08 (s, 1H, CHO), 8.22 (s, 1H, pyrazole H), 7.79–7.83 (m, 4H, 1 and 3-Ar-H), 7.45–7.48 (m, 2H, 3-Ar-H); ^13^C-NMR (CDCl_3_) δ: 183.6, 152.8, 138.8, 136.0, 133.8 (q, *J* = 33.8 Hz), 129.1, 128.5, 126.1, 125.8, 125.3, 123.7, 122.6, 121.9 (q, *J* = 271.8 Hz); IR (KBr) ν: 3107, 3060, 2845, 1695, 1600, 1529, 1401, 1314, 1130, 858, 719 cm^-1^; Anal. Calcd. for C_17_H_8_Cl_3_F_3_N_2_O: C, 48.66; H, 1.92; N, 6.68. Found: C, 48.77; H, 2.03; N, 6.60%.

*1-[(2,6-Dichloro-4-trifluoromethyl)phenyl]-3-(3-chlorophenyl)-1H-pyrazole-4-carbaldehyde* (**6c**): White solid, m.p. 105–106 °C; ^1^H-NMR (CDCl_3_) δ: 10.09 (s, 1H, CHO), 8.21 (s, 1H, pyrazole H), 7.83–7.91 (m, 3H, 1 and 3-Ar-H), 7.31–7.62 (m, 3H, 3-Ar-H); ^13^C-NMR (CDCl_3_) δ: 183.6, 152.8, 137.8, 136.0, 159.6, 133.8 (q, *J* = 33.8 Hz), 129.1, 128.5, 128.1, 126.1, 125.8, 125.2, 123.6, 122.7, 121.8 (q, *J* = 271.8 Hz); IR (KBr) ν: 3106, 3062, 2840, 1696, 1602, 1530, 1403, 1310, 1134, 856, 719 cm^-1^; Anal. Calcd. for C_17_H_8_Cl_3_F_3_N_2_O: C, 48.66; H, 1.92; N, 6.68. Found: C, 48.81; H, 1.90; N, 6.80%.

*1-[(2,6-Dichloro-4-trifluoromethyl)phenyl]-3-(4-bromophenyl)-1H-pyrazole-4-carbaldehyde* (**6d**): White solid, m.p. 116–118 °C; ^1^H-NMR (CDCl_3_) δ: 10.08 (s, 1H, CHO), 8.22 (s, 1H, pyrazole H), 7.74–7.79 (m, 4H, 1 and 3-Ar-H), 7.61–7.64 (m, 2H, 3-Ar-H); ^13^C-NMR (CDCl_3_) δ: 183.6, 152.8, 138.8, 136.0, 134.6, 133.8 (q, *J* = 33.8 Hz), 129.1, 128.5, 125.8, 125.3, 123.5, 122.6, 121.9 (q, *J* = 271.8 Hz); IR (KBr) ν: 3108, 3063, 2841, 1697, 1604, 1532, 1407, 1315, 1131, 858, 719 cm^-1^; Anal. Calcd. for C_17_H_8_BrCl_2_F_3_N_2_O: C, 44.00; H, 1.74; N, 6.04. Found: C, 43.87; H, 1.79; N, 5.96%.

*1-[(2,6-Dichloro-4-trifluoromethyl)phenyl]-3-(3-bromophenyl)-1H-pyrazole-4-carbaldehyde* (**6e**): White solid, m.p. 110–112 °C; ^1^H-NMR (CDCl_3_) δ: 10.09 (s, 1H, CHO), 8.22 (s, 1H, pyrazole H), 7.79–7.83 (m, 4H, 1 and 3-Ar-H), 7.59–7.62 (m, 1H, 3-Ar-H), 7.35–7.38 (m, 1H, 3-Ar-H); ^13^C-NMR (CDCl_3_) δ: 182.9, 153.8, 139.0, 136.7, 134.2, 133.8 (q, *J* = 33.8 Hz), 129.1, 128.5, 126.1, 125.9, 125.3, 123.7, 123.3, 122.6, 121.8 (q, *J* = 271.8 Hz); IR (KBr) ν: 3106, 3062, 2840, 1695, 1602, 1530, 1401, 1313, 1132, 856, 719 cm^-1^; Anal. Calcd. for C_17_H_8_BrCl_2_F_3_N_2_O: C, 44.00; H, 1.74; N, 6.04. Found: C, 43.70; H, 1.83; N, 6.36%.

*1-[(2,6-Dichloro-4-trifluoromethyl)phenyl]-3-(4-methoxyphenyl)-1H-pyrazole-4-carbaldehyde* (**6f**): White solid, m.p. 75–77 °C; ^1^H-NMR (CDCl_3_) δ: 10.08 (s, 1H, CHO), 8.20 (s, 1H, pyrazole H), 7.76–7.79 (m, 4H, 1 and 3-Ar-H), 7.00–7.03 (m, 2H, 3-Ar-H), 3.87 (s, 3H, OCH_3_); ^13^C-NMR (CDCl_3_) δ: 183.2, 160.4, 154.5, 136.1, 134.9, 133.8 (q, *J* = 33.8 Hz), 130.0, 128.2, 125.8, 125.7, 122.8, 121.9 (q, *J* = 271.6 Hz), 113.9, 55.0; IR (KBr) ν: 3110, 3060, 2963, 2850, 1695, 1600, 1530, 1403, 1312, 1130, 858, 719 cm^-1^; Anal. Calcd. for C_18_H_11_C_l2F3_N_2_O_2_: C, 52.07; H, 2.67; N, 6.75. Found: C, 52.10; H, 2.65; N, 6.78%.

*1-[(2,6-Dichloro-4-trifluoromethyl)phenyl]-3-(4-trifluoromethylphenyl)-1H-pyrazole-4-carbaldehyde* (**6g**): White solid, m.p. 88–90 °C; ^1^H-NMR (CDCl_3_) δ: 10.11(s, 1H, CHO), 8.25 (s, 1H, pyrazole H), 8.01–8.03 (m, 2H, 3-Ar-H), 7.74–7.83 (m, 4H, 1 and 3-Ar-H); ^13^C-NMR (CDCl_3_) δ: 183.2, 151.8, 148.3, 139.1, 137.8, 136.9, 135.0, 133.9 (q, *J* = 33.8 Hz), 129.8 (q, *J* = 32.6 Hz), 129.0, 126.1, 123.7 (q, *J* = 270.1 Hz), 122.6, 121.9 (q, *J* = 271.8 Hz); IR (KBr) ν: 3110, 3060, 2842, 1695, 1600, 1528, 1400, 1313, 1130, 858, 719 cm^-1^; Anal. Calcd. for C_18_H_8_C_l2F6_N_2_O: C, 47.71; H, 1.78; N, 6.18. Found: C, 47.84; H, 1.85; N, 6.23%.

*1-[(2,6-Dichloro-4-trifluoromethyl)phenyl]-3-(4-nitrophenyl)-1H-pyrazole-4-carbaldehyde* (**6h**): Yellowish solid, m.p. 206–208 °C; ^1^H-NMR (CDCl_3_) δ: 10.12 (s, 1H, CHO), 8.33–8.36 (m, 2H, 3-Ar-H), 8.27(s, 1H, pyrazole H), 8.13–8.15 (m, 2H, 3-Ar-H), 7.82 (s, 2H, 1-Ar-H); ^13^C-NMR (CDCl_3_) δ: 183.2, 151.8, 148.3, 139.1, 137.8, 136.9, 135.0, 133.8 (q, *J* = 33.8 Hz), 129.6, 126.1, 123.7, 122.6, 121.9 (q, *J* = 271.6 Hz); IR (KBr) ν: 3106, 3062, 2843, 1695, 1602, 1530, 1403, 1310, 1132, 858, 719 cm^-1^; Anal. Calcd. for C_17_H_8_C_l2F3_N_3_O_3_: C, 47.47; H, 1.87; N, 9.77. Found: C, 47.56; H, 1.93; N, 9.62%.

*1-[(2,6-Dichloro-4-trifluoromethyl)phenyl]-3-(6-methoxy-naphthalen-2-yl)-1H-pyrazole-4-carb-aldehyde* (**6i**): White solid, m.p. 144–146 °C; ^1^H-NMR (CDCl_3_) δ: 10.19(s, 1H, CHO), 8.25 (s, 1H, pyrazole H), 7.80–7.92 (m, 5H, 1 and 3-Ar-H), 7.19–7.23 (m, 3H, 3-Ar-H), 3.94 (s, 3H, OCH_3_); ^13^C- NMR (CDCl_3_) δ: 183.2, 158.2, 155.0, 136.4, 134.9, 134.7, 133.8 (q, *J* = 33.8 Hz), 129.7, 128.3, 128.2, 127.0, 126.4, 125.8, 125.4, 123.5, 122.1, 121.9 (q, *J* = 271.6 Hz), 119.2, 105.4, 55.0; IR (KBr) ν: 3108, 3062, 2963, 2840, 1695, 1600, 1528, 1400, 1313, 1130, 858, 719 cm^-1^; Anal. Calcd. for C_22_H_13_Cl_2_F_3_N_2_O_2_: C, 56.79; H, 2.82; N, 6.02. Found: C, 56.65; H, 2.93; N, 6.15%.

## 4. Conclusions

In conclusion, The Vilsmeier cyclization of hydrazones provided an efficient route for the preparation of 1*H*-pyrazole-4-carbaldehydes, and we have successfully developed a general method for the synthesis in good yields of a series of 1-[(2,6-dichloro-4-trifluoromethyl)phenyl]-3-aryl-1*H*-pyrazole-4-carbaldehydes **6a-i** using the Vilsmeier-Haack reagent. The structures of all the title compounds have been confirmed by elemental analysis, ^1^H-NMR and ^13^C-NMR and in addition, the structure of intermediate **5b** was confirmed by X-ray crystallography.
